# Perceptual Auditory Aftereffects on Voice Identity Using Brief Vowel Stimuli

**DOI:** 10.1371/journal.pone.0041384

**Published:** 2012-07-23

**Authors:** Marianne Latinus, Pascal Belin

**Affiliations:** 1 Institute of Neuroscience and Psychology, University of Glasgow, Glasgow, United Kingdom; 2 International Laboratories for Brain, Music and Sound (BRAMS), Université de Montréal and McGill University, Québec, Canada; 3 Department of Psychological and Brain Sciences, Indiana University-Bloomington, Bloomington, Indiana, United States of America; Northwestern University, United States of America

## Abstract

Humans can identify individuals from their voice, suggesting the existence of a perceptual representation of voice identity. We used perceptual aftereffects – shifts in perceived stimulus quality after brief exposure to a repeated adaptor stimulus – to further investigate the representation of voice identity in two experiments. Healthy adult listeners were familiarized with several voices until they reached a recognition criterion. They were then tested on identification tasks that used vowel stimuli generated by morphing between the different identities, presented either in isolation (baseline) or following short exposure to different types of voice adaptors (adaptation). Experiment 1 showed that adaptation to a given voice induced categorization shifts away from that adaptor’s identity even when the adaptors consisted of vowels different from the probe stimuli. Moreover, original voices and caricatures resulted in comparable aftereffects, ruling out an explanation of identity aftereffects in terms of adaptation to low-level features. In Experiment 2, we show that adaptors with a disrupted configuration, i.e., altered fundamental frequency or formant frequencies, failed to produce perceptual aftereffects showing the importance of the preserved configuration of these acoustical cues in the representation of voices. These two experiments indicate a high-level, dynamic representation of voice identity based on the combination of several lower-level acoustical features into a specific voice configuration.

## Introduction

Vocalisations are not unique to humans; long before the development of language, vocalisations were the support of oral communication in numerous animal species [1]–[3]. For humans, voices are of special importance as they are the vector of speech that allows the transmission of thoughts from one individual to another. Yet, voices can also be used to make inferences on the emotional state, gender and identity of an individual, often referred to under the general term of “paralinguistic information” since it is relatively independent from linguistic content [4]. In this regard, voices can be seen as “auditory faces” [4]; yet, paralinguistic processing of voices is under-researched compared to speech and face perception.

All human voices share a similar acoustical organisation: a given voice is characterised by a unique configuration of different acoustical features that partly depends on the anatomical structure of the vocal tract. The two main acoustical features represent the contribution of the sound source, the larynx, and the sound filter, the supralaryngeal vocal tract including the oral and nasal cavities [5], [6]. Generally, the source consists of periodic vibrations of the vocal folds at a given fundamental frequency (f0), perceptually referred to as the “pitch”. The vocal tract acts as a mobile filter that enhances certain harmonics of the f0, the formants, which contributes to the perceived timbre. The f0 and formant frequencies are relatively stable across time when a speaker utters a sustained vowel, but vary with phonation. As they partly reflect the anatomy of the vocal apparatus, these acoustic parameters, as well as their interrelations, are characteristic of a unique voice. Despite within and between speaker variation in these parameters, listeners extract invariant features in the vocal signal to build a representation of a speaker’s identity that can be used to recognize that person from novel utterances [7]–[10]. Although progress has been made in our understanding of voice recognition and, despite much research aiming at identifying acoustic parameters underlying speaker recognition, the format of voice identity representations and their relations to the underlying acoustics remain unclear.

A successful behavioural paradigm providing information regarding the stimulus dimensions important for a given perceptual task is the study of “perceptual aftereffects” [11]. Perceptual aftereffects are illusory percepts apparent on a test stimulus (or probe) induced by a prolonged exposure (i.e., adaptation) to another stimulus (the adaptor); exposure often created via the repeated presentation of the adaptor. For instance, the repeated presentation of a contracted face induces a subsequently presented original face to be perceived as distorted in the other direction (dilated) [12]. Perceptual aftereffects have been used to study different perceptual domains (e.g. motion, form and colour perception). Recently, they have been used to investigate higher-level cognitive functions such as face perception [11]–[15]. While there is substantial evidence for perceptual aftereffects in face and speech perception [16]–[18], the study of perceptual aftereffects in the perception of paralinguistic information of voices is fairly recent [19]–[24]. Note that, in the manuscript, “low-level” refers to processes/representation/information about individual components, or features, of a stimulus such as f0, formant frequencies etc. High-level refers to more abstract processes/representation/information taking into account a combination of features. Using a synthetic voice continuum, Mullennix et al. (1995) reported perceptual aftereffects in the perception of voice gender. In a follow-up experiment using “vocal hybrids” that combined a male f0 and female formants as adaptors, they failed to show aftereffects with either adaptor. This result along with results from other experiments led them to conclude that voice gender was stored in an auditory-based representation rather than in an abstract representation [22], suggesting a relatively low-level (i.e., acoustic-dependent) representation of voice gender. On the contrary, Schweinberger and collaborators (2008) reported high-level aftereffects for voice gender perception: the repeated presentation of a male voice led to a perceptual shift such that gender-ambiguous voices were perceived as more female after adaptation than without adaptation [19], also consistent with results of a recent functional magnetic resonance imaging (fMRI) study [25]. Auditory aftereffects have also been reported in the perception of vocal emotions [23], age [21], [26], and identity [20], [24], [27].

Vocal identity aftereffects have so far been reported in three studies that used different paradigms and led to different results. Identity aftereffects are shifts in the perception of the identity of a probe stimulus after exposure to a given (adaptor) identity [14], [15], [20], [24]. In a first study, Zäske and collaborators (2010) created identity continua by morphing sentences spoken by two speakers familiar to the participants; the identity continua were created by using different weights while morphing the two original voices. They showed identity aftereffects when data were collapsed across the different stimuli composing the identity continua and compared to another adaptation condition rather than to a baseline condition, i.e., in the absence of adaptation [20]. The same group recently reported identity aftereffects with vowel–consonant–vowel (VCV) stimuli [27]; in this latest study, the aftereffects were stronger when the adapting items were the same as the probe (or test) stimulus, suggesting that the aftereffects could partly rely on low-level acoustical information, rather on identity *per se*. In a recent experiment, we have demonstrated high-level aftereffects in the perception of vocal identity using simple vowel stimuli; adaptation with “anti-voice” stimuli induced perceptual changes in the identity of subsequently presented ambiguous voice stimuli [24]. These effects were robust and observed for nearly all individual stimuli of an average voice/individual voice continuum. In summary, previous studies either showed robust aftereffects when the adapting and probe stimuli were similar or relatively weak aftereffects when those were different. Consequently, it is difficult to decipher whether identity aftereffects were due to adaptation to low-level acoustic information or to identity perception *per se*.

We report two experiments to seek new insights into voice identity representations; particularly we wished to 1) test whether identity aftereffects involve a higher-level representation of vocal identity relatively independent of low-level information; and 2) identify the acoustic features, or combination of features, involved in this higher-level voice identity representation. In a first experiment, we examined perceptual aftereffects induced by adaptors that differed from the probe stimuli in vowel quality. We reasoned that such acoustical differences between adaptors and probe would disrupt low-level adaptation effects based on acoustics while preserving adaptation of a higher level, more invariant representation. In a second experiment, we investigated the dependence of these aftereffects on the underlying acoustical structures.

The results demonstrate that voice identity aftereffects rely on a higher-level identity representation relatively independent from low-level information. Moreover, we show that a specific configuration of f0 and formants is essential in leading to those aftereffects suggesting that the adapted voice identity representation is not based on f0 or formant frequencies alone but on their joint combination in a unified configuration.

## Materials and Methods

The University of Glasgow Faculty of Information and Mathematical Sciences (FIMS) ethics committee approved the experiment and it was conducted with the ethical standards laid down in the 1964 Declaration of Helsinki. All participants gave informed written consent and were paid at a standard rate of £6 per hour for their participation.

### Overview

We present two experiments using perceptual aftereffects to investigate voice identity representation. Before taking part in either experiment, participants underwent a voice learning procedure consisting of daily sessions until a pre-determined recognition criterion was reached (see **Voice Learning Procedure**). After the last learning session, they first performed a 2-alternative forced-choice (AFC) identification task on vowel stimuli drawn from identity continua created by morphing the learned voices with each other (see **Morphing Technique**). This first part of the experiments was run in the absence of adaptation and serves to establish a baseline. After this baseline block, participants partook in different adaptation blocks in which they performed the same identification task after within-trial adaptation. In each trial, participants passively listened to the repeated presentation of an adaptor (5 different vowels), before classifying the identity of a subsequently presented probe stimulus.

### General Methods

#### Stimuli and procedure

All voice samples used in the experiments were from male French-Canadian speakers and drawn from a database of high-quality recordings of the Voice Neurocognition Laboratory (VNL, http://vnl.psy.gla.ac.uk). All stimuli were all normalized for energy (Root Mean Square) using *Matlab 7.5.* (The MathWorks, Inc., Natick, Massachusetts, USA).

Stimuli were presented binaurally at a level of 80 dB SPL via headphones (Beyerdynamic DT 770) using *Media Control Functions (MCF)* software (Digivox; Montreal, QC, Canada). Participants sat in a sound-attenuated room and faced a computer screen displaying response boxes containing names associated with voice identities; each box contained one name. Responses were given by clicking on a box on the computer screen with the computer mouse. Data collection was done using *MCF*, and data analysis with *Matlab 7.5*.

#### Voice learning procedure

Prior to testing, participants underwent a learning phase during which they learned to associate 2 (Experiment 1) or 3 (Experiment 2) voices with corresponding names; the 3 names used were: Phil, Ian (Experiment 1) plus Dave in Experiment 2. During the voice learning procedure, participants were exposed to different vocal stimuli produced by the different speakers: stories, words and vowels in French and English. Note that our subjects did not necessarily understand French; however, as the vowels were French, and the task was to pay attention to paralinguistic information carried by the voice, we believe that using both French and English stimuli during training helped our subjects learn the voices.

The learning procedure comprised three parts. First, participants carefully listened to two stories for each voice and learned to associate each voice with a name displayed on a computer screen. Second, they performed a 2- (Experiment 1) or 3- (Experiment 2) alternative-forced choice (AFC) identification task on words and vowels from the same voices. Feedback was provided: after an incorrect response, participants were given the correct answer, and the stimulus was repeated. Finally, their recognition performance was measured in a test phase in which participants performed a 2- or 3-AFC identification task on vowel stimuli only, without feedback. The duration of a voice learning session was approximately 20 minutes and participants performed daily until their performance at the final task reached a pre-determined criterion (voice identity classification above 75% (Exp.1) or 66% (Exp.2) correct). On average, training lasted 2.6 days [range: 1–3 days] in Experiment 1, and 6.4 days [5–10 days] in Experiment 2. After the last training session, participants took part in the experiments during which only vowels were used.

#### Morphing technique

**Figure 1:**
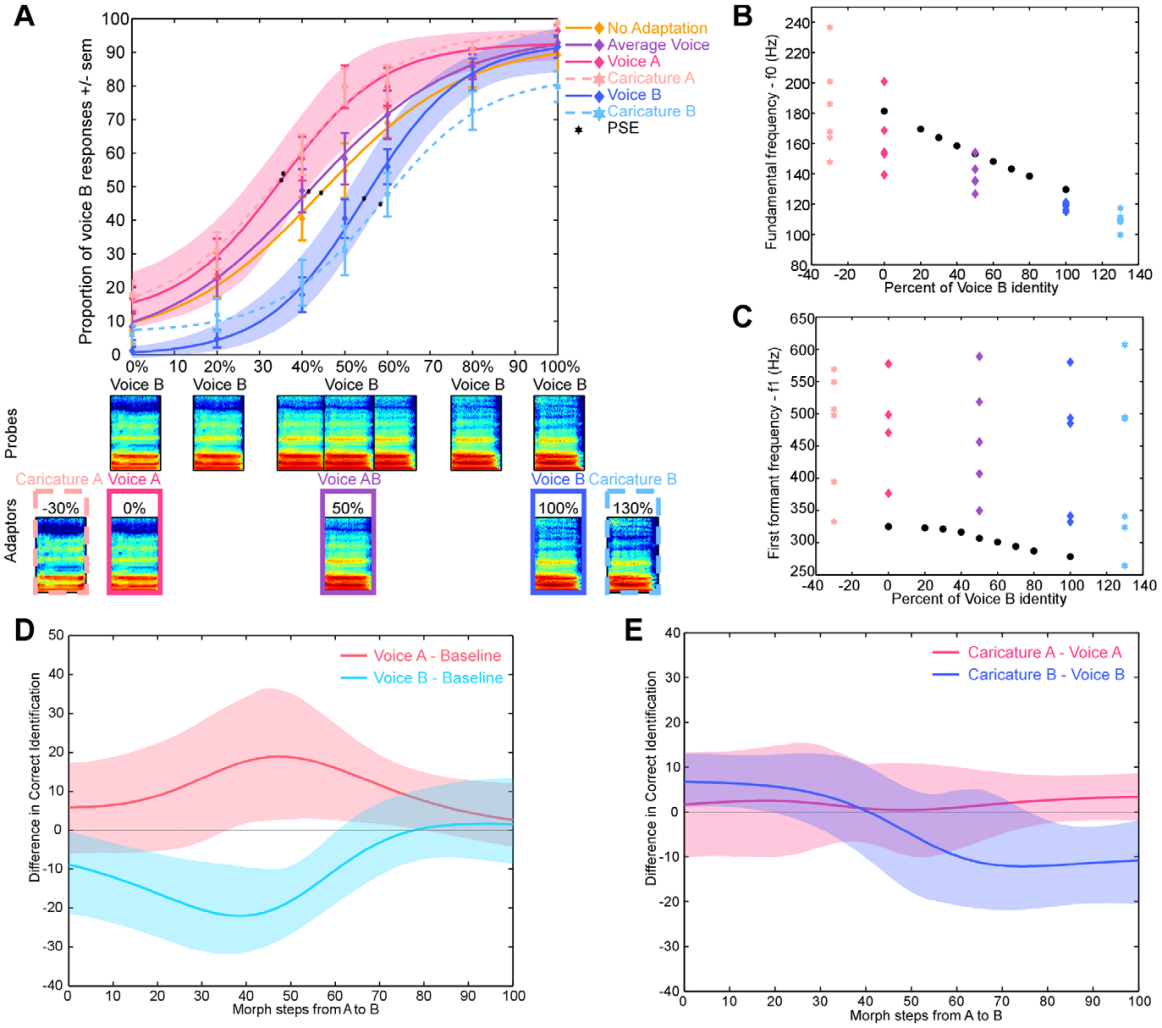
Experiment 1. Results and Acoustical measures (A). Effects of adaptation on voice identification. Shaded areas indicate the bootstrapped 95% confidence intervals (CI_95%_) of the fit to performance after adaptation to voice A and B. Bottom panel: Spectrogram of one vowel continuum (/a/) from voice A to voice B. x-axis: time (0–670 ms), y-axis: frequency (0–6000 Hz). (B) Fundamental frequency. (C) First formant frequency of the different vowels. Black circles indicate stimuli for the A–B continuum for the vowel/u/. Purple triangle: voice AB adaptors. Pink: voice A (star) or caricature of voice A (triangle) adaptors. Blue: voice B (star) or caricature (triangle) adaptors. (D, E). Statistical analysis of Experiment 1. CI_95%_ (shaded area) of the differences in correct recognition performance (line) for each pair of conditions. x-axis: morph steps of the probe stimulus between voices A and B. Morph steps for which the y = 0 line is not contained in the shaded area represent significant performance differences between adaptation conditions. (D) Adaptation to voice A, voice B versus baseline condition. Note that adaptation to voice A and voice B leads to a perceptual change mostly in the identity-ambiguous region (30 to 70%) of the continuum. (E) Adaptation to voice A versus adaptation to caricature A and adaptation to voice B versus adaptation to caricature B. Note that in the identity-ambiguous region of the continuum no differences are seen between adaptation to original voices and caricatures.

Voice identity continua were generated by morphing one voice with another using STRAIGHT (Speech Transformation and Representation by Adaptive Interpolation of weiGHTed spectrogram) [28]. STRAIGHT performs an instantaneous pitch-adaptive spectral smoothing in each stimulus to separate the contributions of the glottal source (including f0) vs. supra-laryngeal filtering (distribution of spectral peaks, including the first formant, F1 [5]) to the voice signal. Voice stimuli are decomposed by STRAIGHT into five parameters: f0, i.e., the perceived pitch, formant frequency, duration (ms), spectro-temporal density and aperiodicity; each parameter can be independently manipulated. In each stimulus, we manually identified time-frequency landmarks at the start and end of each vowel sound to be put in correspondence across voices. Morphed stimuli were then generated by re-synthesis based on the interpolation (linear for time and aperiodicities; logarithmic for f0, formant frequency and spectro-temporal density) of these time-frequency landmark templates. Each stimulus of a continuum between voices A and B was generated using different values of a weight parameter X: a morphed stimulus contains X% of information of voice A and (100-X)% of information of voice B. Values of X between 0% and 100% correspond to morphed stimuli intermediate between A and B, whereas negative values of X correspond to caricatures of A relative to B, and values of X greater than 100% correspond to caricatures of B relative to A (Figure 1A, 1B).

#### Statistical analysis

Data were analysed using a program written in *Matlab 7.5.* Percentage correct data was fitted with a logistic function with 4 free parameters: the minimum and maximum y-values, the x-value at the centre of symmetry of the curve (the point of subjective equality – PSE) and the slope of the curve at the PSE. We used a bootstrap technique (10,000 re-samplings) [29] to assess the statistical significance of the effects and to compute data-driven confidence intervals. Each bootstrap sample consisted of a sampling from the participants “with replacement”, i.e., a participant could be present more than once in each sample, according to standard bootstrapping procedures. Due to our paired design, when a subject was selected randomly, results from all his conditions were included in that sample. For each condition, we averaged the data across participants and fitted this average with the logistic function, then saved the regression parameters. We also computed differences between conditions at this stage. For each condition, we repeated the process 9999 times, leading to a distribution of 10,000 bootstrapped estimates of the fits, and a distribution of fit differences among conditions. Finally, we computed the 95% confidence intervals of fit parameters and the fit differences. A difference is significant at a given continuum step if its confidence interval at that step excludes 0. In Experiment 1, two participants were removed from statistical analyses because their responses were improperly fitted by a logistic function (R^2^<0.4); no subjects were excluded from the analysis in Experiment 2.

### Experiment 1: Methods

#### Participants

Sixteen normal listeners (6 males; mean age: 25 years +/−6.5) with no hearing problems were recruited from the undergraduate population of Glasgow University with no maternal language restrictions.

#### Stimuli and design

Stimuli were sustained French vowels (/a/,/é/,/è/,/o/,/u/and/U/). Two male voices were morphed together with values of a weight parameter X (see **Morphing Technique**) varying between −30% and 130% in 10% steps to create a continuum ranging from a caricature of A (X = −30%), to voice A (X = 0%) then voice B (X = 100%) and a caricature of B (X = 130%); morphed continua were created for the six different vowels (Figure 1B, C) leading to the creation of six identity continua.

After the last learning session, participants first performed a 2-AFC identification task on probe stimuli in the absence of adaptation (baseline, 42 trials). They then performed the same task on the same probe stimuli following within-trial adaptation under 5 different adaptation conditions.

Probe stimuli were drawn from the six identity continua for values of X = 0%, 20%, 40%, 50%, 60%, 80% and 100% (7 probe stimuli, Figure 1A). Adaptors were of 5 different kinds: voice A, voice B, adaptor AB (X = 50%) and the caricatures of voice A and B (Figure 1). Within-trial adaptation was induced by the presentation of 5 different vowels of an adaptor type (vowel average duration: 1110 ms [700–1360]), with a 100 ms interstimulus interval (ISI); hence, the time spent adapting to the stimuli was about 5500 ms per trial. A 2-AFC identification task was then performed on a probe stimulus (average duration: 1110 ms [824–1283]) presented after a 5 s silent interval; the probe vowel was always different from the ones used to produce adaptation. The different adaptation conditions (i.e., to the different kinds of adaptors) were run in separate blocks of 42 trials; none of the vowels was repeated within a trial to ensure that only identity was adapted [19]. The order of the adaptation blocks was counterbalanced across participants.

### Experiment 2: Methods

#### Participants

Thirteen normal listeners (5 males; mean age: 22.5 years +/−3.7) with no hearing problems were recruited from the undergraduate population of Glasgow University with no maternal language restrictions.

#### Stimuli and design

Stimuli were sustained French vowels (/a/,/é/,/è/,/o/,/u/and/U/) uttered by three speakers (A, B, C). The probe stimuli were drawn from identity continua resulting from the morphing of all possible pairs of voices, e.g., voice A with voice B and voice B with voice C (AB, AC, BC; see **Morphing Technique**), and corresponded to stimuli with values of X ranging from 5% and 95% in 15% steps (7 probes). These three identity continua were created for the 6 different vowels resulting in 18 different continua. Subjects were presented with two of the three identity continua, chosen randomly amongst the 3 possible. For a given subject, the two different continua (e.g., AB and BC) were always run in different blocks, so that participants always performed a 2-AFC identification task on the stimuli; results, however, are presented pooled across continua. In the rest of the manuscript, for ease of reading, conditions are described under generic terms defined in the context of an AB continuum.

First, participants performed a 2-AFC identification tasks on the probe stimuli in the absence of any adaptation to establish a baseline (84 trials). Second, participants performed the same 2-AFC identification tasks after within-trial adaptation.

Adaptors were created using STRAIGHT by applying different morphing weight to the f0 and formants frequencies parameters. One set of adaptors was created by applying a weight of 1/2 to the formant parameters (thus averaging them between the two learned identities), while the f0 had a weight of 0 (voice A) or 1 (voice B); these adaptors are referred to as the “f0 adaptors” or adaptors f0_A_/formant_AB_ and f0_B_/formant_AB_. Another set of adaptors was created by applying a weight of 1/2 to the f0 (thus averaging f0 values between the two learned identities) and a weight of 0 or 1 to the formant parameters (thus selecting formant frequencies of either A or B); these adaptors are referred to as “formant adaptors” or f0_AB_/formant_A_ and f0_AB_/formant_B_. Adaptation was induced by the presentation of 5 different vowels from an adaptor set (vowel duration: 670 ms), with a 100 ms ISI; hence, the time spent adapting to the stimuli was about 3750 ms per trial. A 2-AFC identification task was then performed on a probe stimulus (duration: 670 ms) presented after a 1 s silent interval; the probe vowel was always different from the ones used to produce adaptation. The different adaptation conditions (i.e., adaptation to “f0 adaptors” or “formant adaptors” for each identity continua) were run in separate blocks of 42 trials; none of the vowels was repeated within a trial to ensure that only identity was adapted [19]. The order of the adaptation blocks was counterbalanced across participants.

## Results

### Experiment 1

In experiment 1, we tested 5 different adaptors: the original voices (A and B), voice AB, and the caricatures of A and B. No specific perceptual shift was expected with adaptor AB as it should bias identity perception equally towards the two end-points of the continuum; aftereffects would thus be cancelled out. Caricatures of A and B were included to quantitatively assess the dependence of identity perceptual aftereffects on acoustical parameters. In face perception, caricatures are shown to differ from the original stimuli in term of most parameters but are perceived as having the same identity [30]. To confirm that voice caricatures were also perceived as the corresponding identity, 5 new subjects were trained with 2 voices (three 20-min sessions) and performed an identification task on the caricatures and the original voices. Results showed that caricatures were categorized as the corresponding original voice a higher proportion of times than the original voices themselves (94%, and 86% respectively – F(4,1) = 21.41, p = 0.01), consistent with observations on facial stimuli [30], [31].

The participants performed a 2-AFC speaker identification task on vowel stimuli drawn from identity continua after a familiarisation period with the two voice identities (see **Materials and Methods**). Without adaptation, identification yielded a classical logistic categorisation function with a steeper slope in the identity-ambiguous portion of the continuum (i.e., 40% to 60% – Figure 1A, orange curve). We repeated this experiment with within-trial adaptation to one of five different adaptors: the original voices A and B, the average voice between A and B (adaptor AB), and the caricatures of A and B (Figure 1A). Note that adaptation trials consisted of five different vowels that also differed from the probe vowel in order to minimize adaptation to low-level acoustical information (Figure 1B, 1C).

Significant shifts of the psychometric function were observed following adaptation to the original voices and the caricatures (Figure 1A, 1D). However, no significant differences (p>0.05) were found between the baseline condition (PSE abscissa ± s.e.m (standard error of mean): x = 45.9±5.1) and adaptation to voice AB (x = 42.5±4). Adapting with voice A resulted in a significant (p<0.05, Figure 1D) shift of the psychometric function (x = 39.5±4) as compared to baseline: each probe was categorised more often as B. Conversely, adaptation to voice B (x = 55±3.4) also induced a significant shift in the psychometric function as compared to baseline; each probe stimulus was more often categorised as voice A. Adaptation to caricatures induced identity shifts similar to those induced by the original voices (caricature A: x = 41±3.9; caricature B: x = 58.3±4.5; Figure 1E).

Experiment 1 thus provides evidence of perceptual aftereffects with brief vowel stimuli. Simple adaptation to low-level acoustical features cannot explain this result as adaptors consisted of five different vowels all differing from the probe stimulus in their main acoustical characteristics [19], particularly formant frequencies [32]. Consequently, identity was the only characteristic common to the adaptors demonstrating that voice identity representation is relatively independent of low-level acoustic information. Moreover, original voices and caricatures led to similar identity shifts although they differed acoustically from one another (Figure 1B, 1C), again suggesting that the perceptual aftereffects observed had little dependence on low-level acoustical information. Altogether these observations indicate the involvement of a dynamic, high-level representation of voice identity, not directly dependent on acoustical properties [19].

To further test for an abstract representation of voice identity, and to investigate the acoustical parameters that would underlie such a representation, we designed a second experiment in which we manipulated the spectral content of the adaptors. In Experiment 2, two sets of adaptors were generated: f0 adaptors and formant adaptors (see **Materials and Methods**). We reasoned that if voice recognition relied primarily on f0, larger aftereffects should be observed for f0 adaptors – that retained original f0 values of the learned voice identities – than for formant adaptors that both had intermediate f0 values. Conversely, if the crucial information for recognising a speaker lay in the formant frequencies, the formant adaptors would induce stronger aftereffects than the f0 adaptors that were characterized by intermediate formant frequencies. Observing no aftereffects would indicate that the adapted voice identity representation relies on a combination of both fundamental and formant frequencies.

### Experiment 2

**Figure 2:**
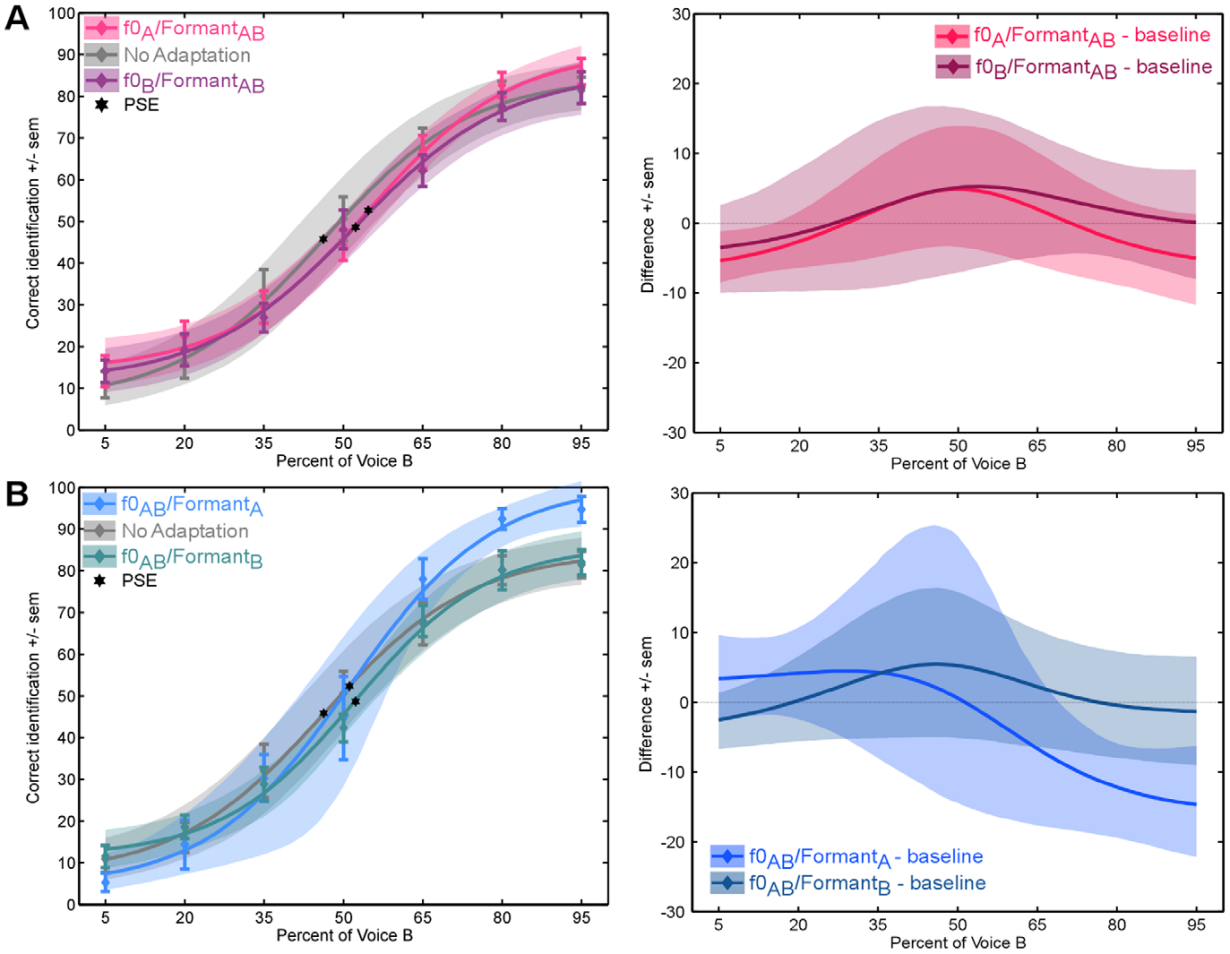
Adaptation to formant and fundamental frequencies. (A) Aftereffects induced with the fundamental frequency adaptor (f0_A_/formant_AB_). Right panel: statistical analysis, differences between each adaptation condition and baseline (i.e., no adaptation). Shaded area: 95% confidence interval. (B) Aftereffects induced with the formant adaptor (f0_AB_/formant_A_). Right panel: statistical analysis, differences between each adaptation condition and baseline (i.e., no adaptation). Shaded area: 95% confidence interval.

Thirteen adult listeners performed a 2-AFC identification task on probe stimuli drawn from an identity continuum with or without adaptation (baseline). There were two sets of adaptors per identity continuum: the f0 adaptors and the formant adaptors. Without adaptation, identification yielded a classical logistic categorisation function with a steeper slope in the identity-ambiguous portion of the continuum (i.e., 40% to 60% – Figure 2A, 2B, grey curve). No differences were seen between the baseline conditions (x = 48.7±3.5) and adaptation to the f0 adaptors (f0_A_/formant_AB_: x = 54.4±1.8, f0_B_/formant_AB_: x = 52.8±3– Figure 2A) or the Formants adaptors (f0_AB_/formant_A_: x = 48.2±3.7, f0_AB_/formant_B_: x = 53.1±2.4– Figure 2B).

## Discussion

We report two experiments that used perceptual aftereffects to provide new insights into the representation of voices. In the first experiment, robust perceptual aftereffects relatively independent of the underlying acoustical properties of the vowels used were shown even with brief vowel stimuli. In the second experiment, we failed to observe identity aftereffects when only the formant frequencies or f0 were that of the original voices, indicating that the adapted voice identity representation involves a combination of both pitch and timbre cues.

In Experiment 1, we found that after hearing a small series of vowels spoken by speaker A, listeners were more likely to categorize an identity-ambiguous vowel from an A–B voice continuum as produced by speaker B, and *vice versa*. Experiment 1 thus confirmed the existence of voice identity aftereffects [20] even for brief vowel stimuli: short exposure to a given voice induced a shift away from the adapting identity. The effect was strong and consistent, showing significant differences at nearly all morph steps of the A–B continuum, even compared to the baseline condition (Figure 1A, 1D). This aftereffect is analogous to a similar phenomenon known for faces [12]–[14]. It can be interpreted as a local distortion of the perceptual “voice space” caused by repetition of a particular identity, enhancing the perceptual distance from other identities. This suggests a highly plastic, history-dependent cerebral representation of identity, consistent with reports of neuronal adaptation to voice identity in the temporal lobe of humans and macaques [3], [9], [33].

Aftereffects were of similar magnitude when adaptors consisted of voice identity caricatures. Caricatures are interesting to test because they differed physically from the natural voices in both formant and fundamental frequencies (Figure 1B, 1C), but have stronger perceptual identities than the original voices. Caricatures have previously been used in order to test the dependence of auditory perceptual aftereffects on low-level acoustic information. Bestelmeyer et al. (2010) expected caricatures to lead to stronger aftereffects than original stimuli if perceptual aftereffects in vocal affect perception had been driven by low-level information; similar adaptation magnitude between caricatures and natural voices was interpreted as reflecting high-level perceptual aftereffects [23]. However, as caricatures differed acoustically from the continuum endpoints, if adaptation was induced by low-level acoustic information, no perceptual aftereffects would be expected [22]. In our experiment, although caricatures were created by exaggerating the features of the original voices with respect to one another rather than to a neutral stimulus, they were still better recognised than the original stimuli, consistent with previous reports in face perception [30], [31]. Our results are unlikely to have been driven by low-level information, due to the adaptation sequence used in which the stimuli were all different as well as different from the probe stimuli. Consequently, we interpreted the lack of difference between caricatures and original voices as reflecting a categorical representation of voice identity: if a voice is recognised as identity A, regardless of the identity strength, it will induce similar identity aftereffects, consistent with the results of an fMRI study of voice identity perception [10].

Perceptual aftereffects can be used in order to determine the stimulus dimensions relevant to perform a perceptual task. In Experiment 2, we used perceptual aftereffects in order to test the role of pitch and formant cues in voice recognition. To that purpose we manipulated the spectral content of adaptors and created two sets of adaptors: f0 and formant adaptors. While in f0 adaptors, the f0 was kept equal to that of the original voices and the formant frequencies were that of the adaptation-neutral AB voice, in the formant adaptors, the formant frequencies were that of the original voices. We failed to show any effect of adaptation when only one of these parameters was preserved in the stimuli. This absence of aftereffects, combined with the presence of aftereffects in Experiment 1, demonstrates that both are crucial in representing voices. This suggests that voice identity is represented via a combination of the spectral information produced by the source and filter aspect of the vocal tract, consistent with previous reports [24], [34].

Importantly, while both caricatures and f0 and formant adaptors physically differed from the continuum’s endpoints, only caricatures induced strong perceptual aftereffects. The important difference between the two adaptors lies in the fact that caricatures retained the configuration of acoustical cues of the original voices, allowing them to be perceived as from the same identity, while this configuration is disrupted in the f0 and formant adaptors. This suggests not only that identity aftereffects are not driven by low-level acoustical information but also that the representation of voice identity indeed relies on a specific combination of acoustic features characteristic of a given voice and is not just dependent on a single feature in isolation.

These two experiments demonstrate robust aftereffects in the domain of vocal identity using brief stimuli. The aftereffects were relatively independent from low-level acoustic features: similar aftereffects were observed for caricatures and original voice adaptors, while adaptors with a modification of either pitch or timbre failed to produce aftereffects. Our results suggest a representation of voice identity relatively abstracted from exemplar-specific acoustical features and sufficiently plastic to be affected by hearing a few vowels from another voice. Moreover, they demonstrate that the correct configuration of the f0 and the formant frequencies is essential in representing voice identity.

## References

[1] Belin P (2006). Voice processing in human and non-human primates.. Philos Trans R Soc Lond B Biol Sci.

[2] Insley SJ (2000). Long-term vocal recognition in the northern fur seal.. Nature.

[3] Petkov CI, Kayser C, Steudel T, Whittingstall K, Augath M (2008). A voice region in the monkey brain.. Nat Neurosci.

[4] Belin P, Fecteau S, Bedard C (2004). Thinking the voice: neural correlates of voice perception.. Trends Cogn Sci.

[5] Ghazanfar AA, Rendall D (2008). Evolution of human vocal production.. Curr Biol.

[6] Latinus M, Belin P (2011). Human voice perception.. Curr Biol.

[7] Kreiman J, Johnson K, Mullenix J (1997). Listening to voices: theory and practice in voice perception research..

[8] Papcun G, Kreiman J, Davis A (1989). Long-term memory for unfamiliar voices.. J Acoust Soc Am.

[9] Formisano E, De Martino F, Bonte M, Goebel R (2008). “Who” is saying “what”? Brain-based decoding of human voice and speech.. Science.

[10] Latinus M, Crabbe F, Belin P (2011). Learning-Induced Changes in the Cerebral Processing of Voice Identity.. Cereb Cortex.

[11] Yamashita JA, Hardy JL, De Valois KK, Webster MA (2005). Stimulus selectivity of figural aftereffects for faces.. J Exp Psychol Hum Percept Perform.

[12] Webster MA, MacLin OH (1999). Figural aftereffects in the perception of faces.. Psychon Bull Rev.

[13] Webster MA, Kaping D, Mizokami Y, Duhamel P (2004). Adaptation to natural facial categories.. Nature.

[14] Leopold DA, O'Toole AJ, Vetter T, Blanz V (2001). Prototype-referenced shape encoding revealed by high-level aftereffects.. Nat Neurosci.

[15] Rhodes G, Jeffery L (2006). Adaptive norm-based coding of facial identity.. Vision Res.

[16] Ades AE, Wales RJ, Walker E (1976). New approaches to language mechanisms..

[17] Samuel AG (1986). Red herring detectors and speech perception: in defense of selective adaptation.. Cogn Psychol.

[18] Holt LL (2006). The mean matters: effects of statistically defined nonspeech spectral distributions on speech categorization.. J Acoust Soc Am.

[19] Schweinberger SR, Casper C, Hauthal N, Kaufmann JM, Kawahara H (2008). Auditory adaptation in voice perception.. Curr Biol.

[20] Zaske R, Schweinberger SR, Kawahara H (2010). Voice aftereffects of adaptation to speaker identity.. Hear Res.

[21] Zaske R, Schweinberger SR (2011). You are only as old as you sound: Auditory aftereffects in vocal age perception.. Hear Res.

[22] Mullennix JW, Johnson KA, Topcu-Durgun M, Farnsworth LM (1995). The perceptual representation of voice gender.. J Acoust Soc Am.

[23] Bruckert L, Bestelmeyer P, Latinus M, Rouger J, Charest I (2010). Vocal attractiveness increases by averaging.. Curr Biol.

[24] Latinus M, Belin P (2011). Anti-voice adaptation suggests prototype-based coding of voice identity.. Frontiers in Psychology 2.

[25] Charest I, Pernet C, Latinus M, Crabbe F, Belin P (2012). Cerebral Processing of Voice Gender Studied Using a Continuous Carryover fMRI Design.. Cereb Cortex.

[26] Schweinberger SR, Zaske R, Walther C, Golle J, Kovacs G (2010). Young without plastic surgery: perceptual adaptation to the age of female and male faces.. Vision Res.

[27] Schweinberger SR, Walther C, Zaske R, Kovacs G (2011). Neural correlates of adaptation to voice identity.. British Journal of psychology.

[28] Kawahara H, Masuda-Katsuse I, Cheveigne AD (1999). Restructuring speech representations using a pitch-adaptive time-frequency smoothing and an instantaneous-frequency-based F0 extraction.. Speech Communication.

[29] Wilcox RR (2005). Introduction to Robust Estimation and Hypothesis Testing. Second Edition.; Press EA, editor.. San Diego, CA: Academic Press.

[30] Benson PJ, Perrett DI (1994). Visual processing of facial distinctiveness.. Perception.

[31] Blanz V, O'Toole AJ, Vetter T, Wild HA (2000). On the other side of the mean: the perception of dissimilarity in human faces.. Perception.

[32] Hillenbrand J, Getty LA, Clark MJ, Wheeler K (1995). Acoustic characteristics of American English vowels.. J Acoust Soc Am.

[33] Belin P, Zatorre RJ (2003). Adaptation to speaker's voice in right anterior temporal lobe.. Neuroreport.

[34] Baumann O, Belin P (2010). Perceptual scaling of voice identity: common dimensions for different vowels and speakers.. Psychol Res.

